# Signatures of selection in sheep bred for resistance or susceptibility to gastrointestinal nematodes

**DOI:** 10.1186/1471-2164-15-637

**Published:** 2014-07-30

**Authors:** Kathryn M McRae, John C McEwan, Ken G Dodds, Neil J Gemmell

**Affiliations:** Centre for Reproduction and Genomics, Department of Anatomy, University of Otago, Dunedin, New Zealand; AgResearch, Invermay Agricultural Research Centre, Mosgiel, New Zealand

## Abstract

**Background:**

Gastrointestinal nematodes are one of the most serious causes of disease in domestic ruminants worldwide. There is considerable variation in resistance to gastrointestinal nematodes within and between sheep breeds, which appears to be due to underlying genetic diversity. Through selection of resistant animals, rapid genetic progress has been demonstrated in both research and commercial flocks. Recent advances in genome sequencing and genomic technologies provide new opportunities to understand the ovine host response to gastrointestinal nematodes at the molecular level, and to identify polymorphisms conferring nematode resistance.

**Results:**

Divergent lines of Romney and Perendale sheep, selectively bred for high and low faecal nematode egg count, were genotyped using the Illumina® Ovine SNP50 BeadChip. The resulting genome-wide SNP data were analysed for selective sweeps on loci associated with resistance or susceptibility to gastrointestinal nematode infection. Population differentiation using F_ST_ and Peddrift revealed sixteen regions, which included candidate genes involved in chitinase activity and the cytokine response. Two of the sixteen regions identified were contained within previously identified QTLs associated with nematode resistance.

**Conclusions:**

In this study we identified fourteen novel regions associated with resistance or susceptibility to gastrointestinal nematodes. Results from this study support the hypothesis that host resistance to internal nematode parasites is likely to be controlled by a number of loci of moderate to small effects.

**Electronic supplementary material:**

The online version of this article (doi:10.1186/1471-2164-15-637) contains supplementary material, which is available to authorized users.

## Background

Gastrointestinal nematodes are one of the most serious causes of disease in domestic ruminants worldwide [1, 2]. Production losses due to parasitism are two-fold; the direct cost of anthelmintic treatment and production losses due to ill-thrift and in extreme cases death [3]. In the face of the increasing incidence of anthelmintic resistance and the need to minimise drench residues in animal products, new strategies for control are required [4].

Breeding for host resistance has been shown to be a viable method of nematode control [5]. Host resistance is heritable, with wide variability among individuals, and rapid genetic progress has been demonstrated in both research and commercial flocks [6, 7]. Moreover, computer simulation models have shown that selection for host resistance, using the phenotype low faecal worm egg count, should be stable over a short time frame such as 20 years [8]. This is supported by field data, where it was shown that when gastrointestinal nematodes were exposed to genetically resistant or susceptible sheep over a sustained period of time they showed no evidence of adaptation to their host [9]. These findings support the hypothesis that resistance is determined by many genes each with a relatively small effect [10] and that selection for parasite resistance based on faecal egg count (FEC) is sustainable in the medium to long-term.

With sheep it is possible to manipulate breeding lines to produce strong phenotypic differences, in well-defined pedigrees, in a relatively short space of time. Reduction of variation in genomic regions surrounding a beneficial mutation due to strong and recent selection is known as a “selective sweep”; identification of regions that have undergone selective sweeps can help to reveal genes underlying phenotypic differences. Different statistics pick up different patterns of variation left by selection of a beneficial mutation. Wright’s fixation index (F_ST_) is a single marker test that detects highly differentiated alleles, where positive selection in one area causes larger frequency differences between populations as compared to neutrally evolving alleles. Peddrift [11] is a program that also uses single markers to calculate exact probabilities of allele frequency differences, by using the recorded pedigree structure to take into account minor allele frequencies, genetic drift, founder and sampling effects. Evidence of selection is shown by divergence from the expected distribution (given by a P-value). Unlike F_ST_ and Peddrift, tests based on linkage disequilibrium, such as the extended haplotype homozygosity (EHH) statistic and its derivatives, are dependent on SNP spacing and frequency, as they are multi-marker tests. The integrated haplotype score (iHS) [12] and cross population EHH (XP-EHH) [13] tests are both based on extended haplotype. While iHS detect partial selective sweeps a moderate frequency (~50-80%), XP-EHH detects alleles that have risen to near fixation in one population (>80% frequency), but remain polymorphic in the population as a whole. Studies that search for signatures of selective sweeps tend to use multiple tests as they are largely complementary; iHS and XP-EHH have been used to search for recent positive selection in humans [13, 14], as well as other species such as cattle [15, 16].

Recent advances in genomic technologies have provided new opportunities to detect regions in the sheep genome that have undergone selection. The advent of the SNP50 BeadChip provided 54,241 evenly spaced Single Nucleotide Polymorphisms (SNP) across the sheep genome for association analysis. The chip has already been utilised to map causal mutations for traits controlled by a single locus [17–21] and detect signatures of selection among sheep breeds [22, 23]. The identification of genes or linked markers that have a significant association to parasite resistance could accelerate the genetic improvement of resistance to internal nematodes through marker-assisted selection [24]. Additionally, identification of genes under selection in animals selected for resistance or susceptibility to gastrointestinal parasites will help in our understanding of the biological processes underlying this trait.

The SNP50 BeadChip provides a rapid way to detect regions under selection, which can be further fine-mapped using Sequenom® or other technologies. To this end, lines of sheep that have been selected for resistance, resilience, or susceptibility coupled with high-density genetic maps are a key resource that would enable future marker assisted selection of animals without the need for parasite challenge. Here we utilise data from Romney and Perendale parasite selection lines to conduct whole genomic screens for selection, in the hope of identifying loci, within and between the two breeds, that affect individual host resistance or susceptibility to nematode parasites.

## Results

### Quality control

After quality control (see methods) the final data set consisted of 46,736 SNP for the Romney data set and 48,436 SNP for the Perendale data set. In total 177 Romney (82 high FEC and 95 low FEC) and 146 Perendale (72 high FEC and 74 low FEC) animals passed the quality control. The average MAF of the remaining SNP over all samples was 0.24 (SD = 0.16) in the Romney data set and 0.26 (SD = 0.15) in the Perendale data set.

### Genome-wide analysis

Two analytical methods, F_ST_ [25] and Peddrift [11], were used to detect differentiation between resistant and susceptible animals based on SNP allele frequencies. While F_ST_ is a generic population differentiation statistic, Peddrift is specific to this example in that it was designed to account for structure within the population surveyed.

**Figure 1:**
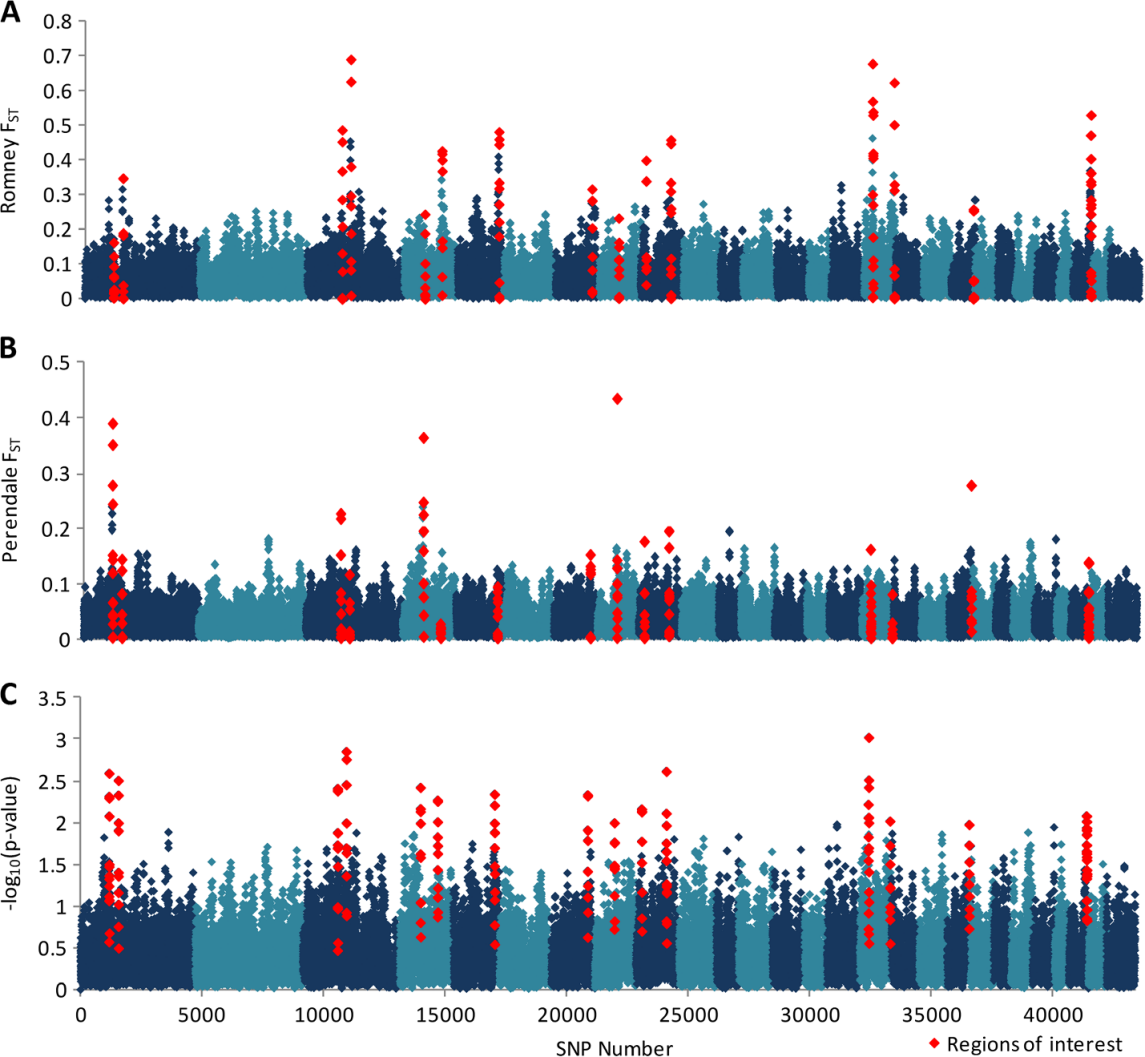
**Genome wide signatures of selection.** A moving window of 5 F_ST_ values between the resistant and susceptible Romney **(A)** and Perendale **(B)** lines. **(C)** A moving average (of 5 SNP) showing the one-tailed probability of the chi-squared distribution of the combined Romney and Perendale Peddrift P-values. Results are expressed as -log_l0_ (significance probability). Regions of interest as defined by WIN5 –log_10_ (combined Peddrift P-values) (Table 1) are shown in red.

As individual SNP may not show a strong signal, a 5-SNP moving average (WIN5) was used to identify regions with strong signatures of selection over multiple SNP, which also reduces noise [26]. The average WIN5 F_ST_ value in the Romney selection lines (Figure 1A) was 0.0567 (SD = 0.0386), while differentiation was lower in the Perendale selection lines (Figure 1B) with an average WIN5 F_ST_ of 0.0299 (SD = 0.0388). A total of 16 genomic regions contained the top 0.1% of markers (Table 1) ranked using WIN5 –log_10_(combined Peddrift P-values) (Figure 1C), with four regions containing genes that have previously been implicated or are candidates for resistance or susceptibility to gastrointestinal nematodes. The first region, on chromosome 1 (region 2), contains the leukocyte surface antigen *CD53*, as well as *DENND2D* and three genes from the chitinase family, acidic mammalian chitinase (*CHIA*), chitinase 3-like 2 (*CHI3L2*) and oviduct-specific glycoprotein (*OVGP1*). Selection was also observed on chromosome 4 (region 5), chromosome 16 (region 14) and chromosome 19 (region 15), containing genes previously implicated in resistance to gastrointestinal nematodes.

**Table 1:** **Genomic regions containing the top 0.1% of SNP, ranked using a moving (5 SNP) average (WIN5) of –log**_**10**_ **(combined Peddrift P-values)**

Region	Chr	Position (Mb)	SNP50 BeadChip SNP	Top SNP	Peak SNP	Peak SNP rank	F _ST_ Peak		PEDDRIFT WIN5 -log _10_ (p-value)			Genes	Candidate gene(s) in region (OARv3.1)
							Romney	Perendale	Romney	Perendale	Combined		
1	1	66.74-67.43	14	4	s43910	5	0.1632	0.3901	0.6799	3.0134	2.5988	1	
2	1	87.38-88.13	10	3	OAR1_93166689	7	0.3475	0.1424	2.1441	1.3027	2.5124	18	*CD53, CHI3L2, CHIA, DENND2D*
3	3	78.72-79.32	12	3	s08153	11	0.4868	0.2270	1.7146	1.6205	2.4161	3	
4	3	98.12-98.51	9	4	OAR3_104545117_X	2	0.6897	0.1148	3.1276	0.7050	2.8569	0	
5	4	44.99-45.39	10	4	OAR4_47833230	10	0.2439	0.3645	0.6647	3.0036	2.4283	2	*RELN*
6	4	83.23-83.69	12	4	OAR4_88693058_X	20	0.4264	0.0256	2.9894	0.3238	2.2755	0	
7	5	95.21-95.69	11	3	OAR5_103935962	14	0.4816	0.0927	2.7849	0.6253	2.3464	2	
8	7	81.54-82.04	9	2	OAR7_89131104	15	0.3167	0.1509	1.8827	1.6553	2.3387	10	
9	8	41.02-41.48	11	1	OAR8_44326031_X	39	0.2327	0.4345	0.9548	2.1834	2.0028	1	
10	9	11.31-11.69	8	3	OAR9_11436829	26	0.3990	0.1750	1.8185	1.2966	2.1687	0	
11	9	66.27-67.09	13	2	OAR9_70612779	4	0.4586	0.1935	1.9607	1.6057	2.6214	0	
12	16	16.67-16.88	7	2	s59518	23	0.6775	0.1604	2.1737	0.9608	2.2246	2	
13	16	17.33-17.7	11	5	s61002	1	0.5391	0.0953	3.2562	0.7822	3.0273	0	
14	16	66.35-66.7	9	1	s54054	35	0.6238	0.0791	2.4545	0.4334	2.0232	4	*NSUN2*
15	19	54.68-55.19	9	1	OAR19_58095077	44	0.2582	0.2780	0.9065	2.1367	1.9812	3	*HRH1*
16	25	39.57-40.32	19	2	OAR25_41988307	31	0.5301	0.1371	2.5086	0.9984	2.0855	1	

The top 5% of SNP were used to define the boundaries of each region (see Materials & Methods). Position is taken from build 3.1 of the sheep genome. The number of SNP50 BeadChip SNP and the number of top SNP (0.1%) are given in each region. Peak SNP is defined as the SNP with the lowest WIN5 –log_10_(combined Peddrift P-value) in the region, and the rank of that SNP is also shown. Maximum F_ST_ and –log_10_ (Peddrift P-value) are shown for each breed. The number of genes in each region, along with candidates (defined as those with a previously reported role in immunity) was taken from Ensembl release 74.

### Investigation of selection sweep on OAR1

In total, 41 extra SNPs were genotyped in region 2; after quality control using the same criteria applied to the SNP50 BeadChip data, 15 of these SNP were used for further analyses. As a consequence of genotyping extra SNP, the peak F_ST_ value in the region increased slightly from 0.3475 (Table 1) to 0.3895.

The LD correlation coefficient r^2^ in region 2 was calculated for each of the selection lines separately (Additional files 1, 2, 3 and 4). All four analyses showed a haplotype block between 12 SNP (Table 2) in region 2. The Romney selection lines showed high linkage disequilibrium (r^2^ > 0.8) within the haplotype block, consistent with selection being imposed on the locus [27].

**Table 2:** **The 11 SNP core haplotype shown by Sweep (v1.1) to be in LD in region 2 (Table** 1 **) on chromosome 1**

SNP ID	Chr	Position	Platform	SNP	Haplotype							
				(Derived/ancestral)	1	2	3	4	5	6	7	8
OAR1_93117979	1	87702641	Sequenom®	T/C	C	C	C	C	C	C	C	T
OAR1_93118073	1	87702735	Sequenom®	C/T	T	T	T	T	T	T	T	C
OAR1_93124341.1	1	87709264	SNP50 BeadChip	T/C	C	C	C	C	C	T	T	T
OAR1_93154178	1	87740396	Sequenom®	A/G	A	G	G	G	G	A	G	A
s50853	1	87751371	Sequenom®	G/C	G	C	C	G	G	C	C	C
OAR1_93166689.1	1	87753155	SNP50 BeadChip	G/T	T	T	T	T	T	T	T	G
OAR1_93219257.1	1	87791568	SNP50 BeadChip	C/T	C	C	C	C	C	C	C	T
OAR1_93226648	1	87801889	Sequenom®	A/G	G	G	G	G	G	G	G	A
OAR1_93231706	1	87807624	Sequenom®	G/T	T	T	T	G	T	T	T	T
OAR1_93231813	1	87807731	Sequenom®	T/C	T	T	T	C	T	C	T	T
s57213.1	1	87826285	SNP50 BeadChip	C/T	C	C	T	C	T	C	C	T
OAR1_93251748	1	87828912	Sequenom®	A/G	-	-	-	-	-	-	-	-
				**Selection line**	**Haplotype frequency**							
				Romney resistant	-	-	-	0.3211	-	-	-	0.6789
				Romney susceptible	-	-	-	0.8841	-	-	-	0.0976
				Perendale resistant	0.0203	-	0.0203	0.6554	0.0338	0.0203	0.0405	-
				Perendale susceptible	-	0.0278	-	0.3264	0.0764	-	-	0.5417

Haplotypes 1–8 were present in either the Romney or Perendale animals, or both. Haplotype frequencies are given for each selection line.

Analysis using Sweep (v1.1) showed that 11 of the 12 above SNP created a haplotype block. In the Romney lines two contrasting haplotypes were observed, denoted 4 and 8 (Table 2). In the Romney lines haplotype 4 was present in 88.4% of the susceptible population, and 32.1% of the resistant population. In the Perendale animals the frequency of haplotype 4 was higher in the resistant animals (65.5%) compared to the susceptible animals (32.5%). There were six additional haplotypes observed in the Perendale selection lines, although they were less frequent (2-8% of the population).

**Figure 2:**
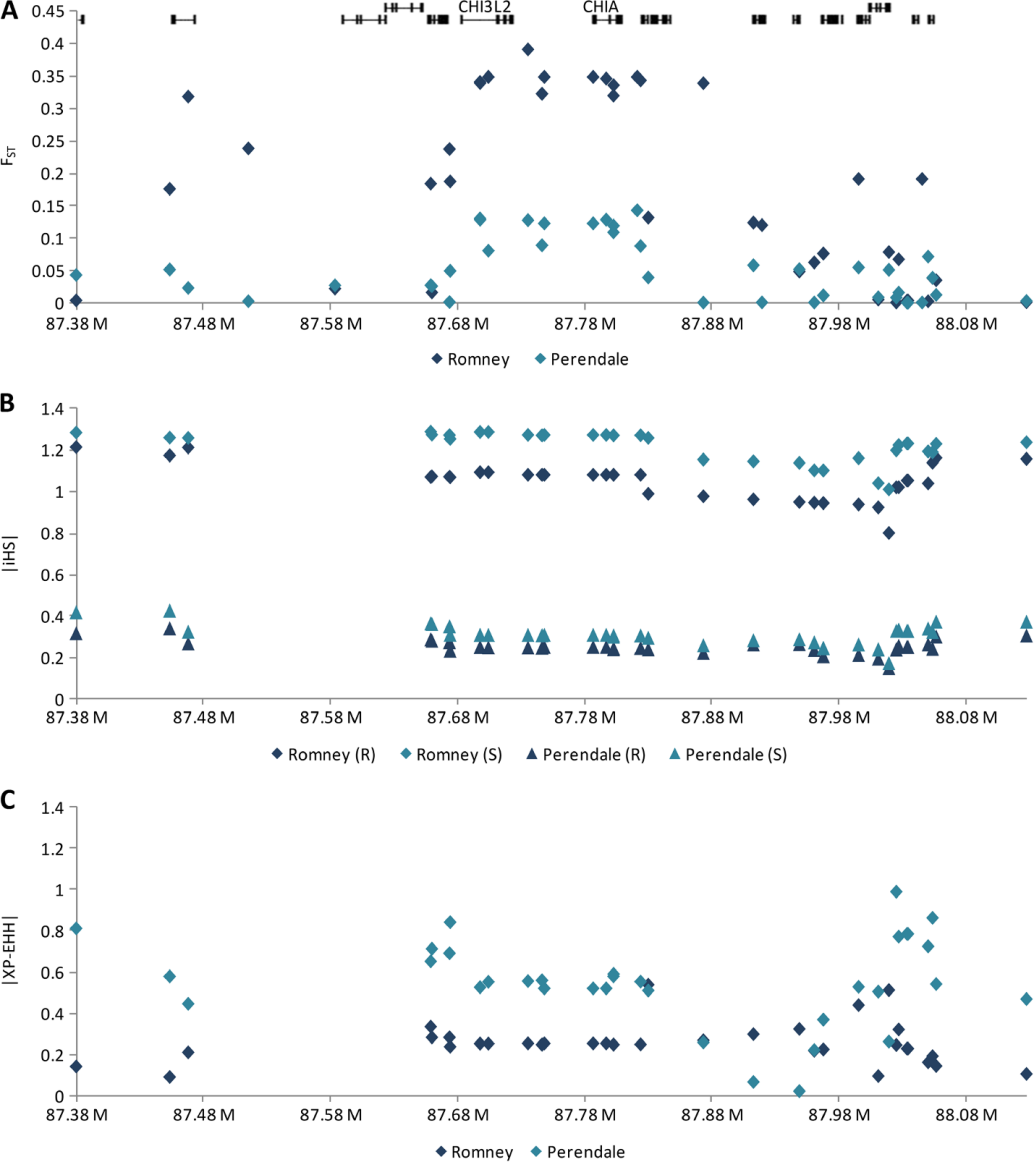
**Signatures of selection observed in region 2 (Table** 1 **) in Romney and Perendale selection lines. (A)** F_ST_ between resistant and susceptible lines, -log P-values from standardised |iHS| **(B)** and |XP-EHH| **(C)** analyses.

The integrated haplotype score (iHS; Figure 2B) [12] and cross-population extended haplotype homozygosity test (XP-EHH; Figure 2C) [13] are designed to uncover selected alleles with higher frequency than expected to their haplotype length. The most significant results were in the Romney susceptible animals where iHS values approached significance (P = 0.0518).

### Sequencing

After examination of the signals of selection in region 2 (Figure 2), the candidate gene *CHIA* (chitinase, acidic) was chosen for exon sequencing. *CHIA* has previously been associated with the control of helminth infection [28]. Other genes in the region include *CD53*, *CHI3L2*, *CHIA*, and *DENND2D*. Sequencing the *CHIA* exons of animals homozygous for both haplotypes showed the presence of several SNP (Additional file 5), however there were no SNP that distinguished the animals of different haplotype or selection line. One SNP at base 1174 of the ovine *CHIA* mRNA could potentially differentiate animals homozygous for haplotype 4 or 8. This, however, would require genotyping in further animals to validate.

## Discussion

Using single-marker tests for differentiation between selection lines, multiple areas were discovered where allele frequency differed between resistant and susceptible lines (Figure 1). This was expected, as variation in complex traits such as resistance to parasites are understood to be controlled by many polymorphisms, each of a small effect [10]. The classic model of a selective sweep involves a beneficial allele being rapidly driven to fixation (‘hard sweep’). However, with complex traits selection may occur through polygenic adaptation, where adaptation occurs by simultaneous selection on variants at many loci. Selection under a polygenic adaptation model would result in modest allele frequency changes across the genome, which may be undetectable using current methods for detecting selection [29]. Despite this, the ‘hard sweep’ and polygenic models are not mutually exclusive, and the alleles with largest effect sizes may sweep to fixation [30]. Polygenic traits will therefore show increased F_ST_ across the genomes, with alleles of a large effect showing increased F_ST_ in that particular region.

Divergent lines of Romney [6, 31] and Perendale [7] sheep were selectively bred for high and low FEC by AgResearch Ltd from 1978 and 1986, respectively (Table 3). One of the aims of this study was to discover if the Romney and Perendale selection lines have utilised the same genetic strategy in developing resistance or susceptibility to internal parasites. Combined Peddrift values were used to define the regions to examine for candidate genes as the test was designed to account for structure within each of the populations surveyed. While peaks were observed in both lines, these were better defined when smoothing, via a 5-SNP window, was applied (Figure 1C).

**Table 3:** **Summary data from Romney and Perendale selection lines** [6, 7]

Trait	Romney		Perendale	
	High	Low	High	Low
Average yearly flock size (rams/ewes)	6/100		4.6/50	
Divergence of log-transformed FEC between lines	2.73		0.85	
Divergence in average BVs between lines	2.83		1.77	
Fold-difference in FEC mean between lines	11.3		4.9	
Heritability	0.28 ± 0.02		0.18 ± 0.03	
Back-transformed FEC means (eggs/g)	3823	339	556	114

It must be noted that the strongest signals of selection were observed in the Romney selection lines, and the strength of the selection would have affected the combined data. As expected, the most extreme values for all statistics in the Romney selection lines were larger than those observed in the Perendale selection lines.

The Perendale lines have not been selected for as long (23 versus 31 years) and the genetic divergence in the selected trait is only half as large (Table 3). The effective population size of the foundation animals is also likely to have had a strong bearing on the differences between the breeds, although this is difficult to determine due to the combination of two separate Romney selection lines. The effective population size (N_e_) for NZ industry Romneys 5 generations ago was estimated as 226 and for Perendale 109, derived from extensive analysis of more than 10,000 New Zealand animals genotyped with the SNP50 BeadChip [32]. As an interbreed of Cheviot and Romney [33] the Perendale animals are also likely to have a higher (N_e_) [34, 35] than a pure breed, which may contribute to the observed pattern in the data. However, since its establishment, the Perendale breed has had a smaller population, which may contribute to low LD between closely spaced markers distances but greater LD between distant markers.

In the regions showing signatures of selection, candidate genes were defined as those with a previously reported role in immunity. We recognise that by examining in detail only those genes with obvious functional links to immunity we have eliminated some genes that could have novel and unexpected effects on the trait concerned, or may contain as yet unidentified features that have an effect separate from the gene itself. However, we believe our approach is a tractable solution, with the data and annotation currently available, and there will be potential to extend this analysis in the future. For example, several regions that appeared to be under selection from our analyses appear to contain no underlying genes (Table 1; Additional file 6). The current annotation of the sheep genome is not as comprehensive as that in humans or even cattle, and these areas cannot be completely dismissed as containing no genes or regulatory elements. This can only be improved following the recent annotation of version 3.1 of the sheep genome by Ensembl (Ensembl release 74). It has also been observed that while some proposed candidates for selection have strong support in the form of a functional mutation with an identified phenotypic effect, often the functional target is unknown [36].

The discovery that the same core haplotype (haplotype 4) in region 2 (Table 2) is observed in both susceptible Romney and resistant Perendale animals does not have an obvious explanation, but could be due to epistatic effects or a recent novel mutation. There was no correlation between haplotype and average estimated FEC breeding value. Following this, there are several possible reasons for the observed differences. It appears that selection in region 2 is primarily occurring in the Romney susceptible line. This is supported by the greater number of haplotypes that were observed in the Perendale selection lines in the Sweep (v1.1) analysis. Sequencing the *CHIA* exons of animals homozygous for both haplotypes showed the presence of several SNP; however none were responsible for the observed haplotype. The observed effect could also be driven by a regulatory element, such as a transcription factor, that could be interacting with a locus or loci in other parts of the genome [37]. In addition, while perhaps not the most likely scenario, a causal mutation in the region could have occurred separately in Perendale and Romneys, on the opposite haplotype block, which would explain the differences observed. Unravelling this, however, is complicated by the fact that the Perendale breed was formed in 1956 by crossing a Cheviot over a Romney, thus half of the Perendale genome is in effect of Romney origin.

Comparison with other studies showed that only two of the regions identified using Peddrift values (Table 1) were contained within a previously identified QTL (Sheep QTLdb [38]). Region 8 overlaps a QTL located on chromosome 7 (CSAP35E–MCM149; OAR7:44,018,971-81,694,614) for resistance to *Haemonchus contortus* infestation in merino sheep [39]. The QTL was not considered by the authors a good candidate for fine-mapping because evidence for the QTL decreased with confirmatory mapping. Region 16 is contained within two suggestive QTL detected on chromosome 25 in a genome scan for for resistance to *Haemonchus contortus* resistance in Romane x Martinik Black Belly backcross lambs [40]. The suggestive QTL, for sex ratio in the adult worm population (0.4-40.7 Mb; OARv2.0) and packed cell volume after second challenge (6.6-44 Mb; OARv2.0), were discovered using linkage analysis with SNP data.

Previously many studies have focussed specifically on chromosomes 3 and 20, which contain interferon gamma (*IFNG*) and the major histocompatibility complex (MHC) region respectively. The SNP50 BeadChip contains four SNP within the IFNG locus (OAR3:151,528,059-151,532,204); the maximum WIN5 –log_10_ (combined Peddrift P-values) in the region was 0.62, which was only slightly higher than the genome-wide average of 0.42. The Romney and Perendale F_ST_ peaks were 0.0505 and 0.0377 respectively, which when compared to the genome wide distributions (Figure 2A & B) is fairly low. Both Romney and Perendale selection lines showed no obvious signals of selection on the other common candidate region, the MHC region on chromosome 20, with a chromosome-wide WIN5 –log_10_ (combined Peddrift P-values) peak of 1.56. While this value is reasonably high when compared to the genome-wide distribution (Figure 1), the highest ranked SNP in the region, going by WIN5 –log_10_ (combined Peddrift P-values), was 167^th^ (OAR20_1876702).

Four regions (Table 1) contained genes that have previously been implicated or are candidates for resistance or susceptibility to gastrointestinal nematodes; OAR 1 (*CD53*, *CHI3L2*, *CHIA* and *DENND2D*), OAR 4 (*RELN*), OAR 16 (*NSUN2*) and OAR 19 (*HRH1*).

The leukocyte surface antigen CD53 contributes to the transduction of CD2-generated signals in T cells and natural killer (NK) cells [41]. NK cells have been shown to produce significant amounts of IL-5, which contributes to eosinophil recruitment in an *in vivo* mouse model of allergic inflammation [42], and may also be involved in T-cell-independent eosinophil recruitment after helminth infections [43]. The CD53 protein forms functional interactions with prominent leukocyte receptors including MHC molecules and the surface of B cells [44], and has been shown to be down-regulated upon stimulation of human neutrophils with TNF-α [45]. In humans CD53 deficiency has been associated with recurrent infectious diseases caused by bacteria, fungi and viruses [46], and polymorphisms in the gene have been associated with regulation of TNF-α levels [47]; up-regulation of *TNF-α* has been observed in the abomasal lymph node of sheep challenged with *T. circumcincta* 5 days after infection [48], and abomasal mucosa of resistant DRB1*1101 carrier lambs at 3 days post infection [49].

Chitinases are a group of digestive enzymes that break down glycosidic bonds in chitin, which is present in fungi and the exoskeletal elements of some animals, including nematodes and arthropods [50]. Mammalian chitinases and chitinase-like proteins are known to be up-regulated and secreted in T_H_2 induced inflammatory responses, including nematode infection [51] suggesting these genes are plausible candidate genes for mediating resistance status.

*CHIA* [52] has previously been associated with the development of the immune response in mammals and control of helminth infection [28]. Induction of *CHIA* is via a T_H_2 specific, IL-13 mediated pathway, and has been implicated in T_H_2 dominated disorders such as asthma [53]. In mice it has been shown that chitin is a recognition element for tissue infiltration by innate immune cells implicated in allergic and helminth immunity (including eosinophils and basophils) and this process can be negatively regulated by a vertebrate chitinase [54]. Despite this, there is no evidence in the literature that *CHIA* has previously been implicated in increased resistance or susceptibility to gastrointestinal parasites in ungulates.

Chitinase-like proteins can bind chitin, however, due to mutations in their active domains they do not have chitinolytic enzyme activity [28]. The chitinase-like molecule, *CHI3L1*, has been shown to be up-regulated in the abomasum of sheep in response to *T. circumcincta* challenge of previously infected animals [55]. *CHIA* expression levels were also examined in the same study, and while expression was observed up-regulation of transcripts was not significant. Expression of *CHI3L2* (UGID: 1230481; http://www.ncbi.nlm.nih.gov/UniGene) has been observed in the abomasum of 18 and 21 week old steers exposed to *Ostertagia ostertagi* [56]. Expression also been observed in the abomasal lymph node of resistant and susceptible Blackface lambs infected with *T. circumcincta* in comparison to sham-infected controls [57]. In human macrophages *CHI3L2* has been found to be upregulated by IL-4 and TGF-β [58].

While the T_H_1/T_H_2 dichotomy has not been proven in sheep, the components involved in response to gastrointestinal parasite infection are typical of a T_H_2 pathway; immunity is associated with the production of T_H_2-associated cytokines, increased numbers of mast cells, peripheral and tissue eosinophilia, and elevated production of multiple antibody isotypes [59–62]. *HRH1* is predominantly expressed on T_H_1 cells, in an IL3-upregulatable manner [63]. Mice lacking *HRH1* had lower percentages of Interferon-γ (*IFNG*)-producing cells, and produced higher levels of antigen-specific IgG1 and IgE. Mice lacking either *HRH1* or *HRH2* tended to have a higher frequency of IL4-producing cells. Jutel *et al*. [63] concluded that histamine secreted from mast cells and basophils potently influences T_H_1 and T_H_2 responses, as well as antibody isotypes, as a regulatory loop in inflammatory reactions. In Blackface lambs challenged over a period of three months with *T. circumcincta*, significantly increased levels of *HRH1* expression in the abomasal lymph node were observed [57].

While the genes *DENND2D*, *RELN* and *NSUN2* do not have obvious roles in immunity, they have previously been reported as being upregulated in susceptible animals. The DENND2D protein was found to be more abundant in genetically susceptible sheep following infection by *H. contortus* [64]. *RELN* was upregulated in Suffolk (susceptible) compared to Texel (resistant) animals three days post infection with *T. circumcincta* [65]. Finally, in a study comparing gene expression in the duodenum following natural infection of lambs from the Perendale selection lines used in this study, *NSUN2* was found to be more highly expressed in susceptible animals [66].

For complex traits, where many loci of small to moderate effect are likely to influence phenotype, the 50,000 SNP available for ovine analysis, which are also biased to high MAF SNP, may not provide enough information. In sheep, single markers were estimated to explain a maximum of 0.48% or 0.08% of the phenotypic variance in FEC following challenge with either *T. colubriformis* or *H. contortus* respectively [10]. It has been suggested in cattle, based on the F_ST_ difference between adjacent loci, that 150,000 evenly spaced SNP would be required to study selective signatures across the bovine genome [67].

In humans, the search for selective sweeps is aided by a large number of densely spaced SNP, with over 3.1 million SNP available from Phase II of the HapMap project (approximately one marker per 1 kb) [68]. Densely spaced SNP give greater power when using statistical tests that rely on linkage disequilibrium (LD), as signals of selection are less likely to be lost. The SNP50 BeadChip, while providing uniform genome-wide coverage, is estimated to have only one marker every 46 kb. Fine-mapping, where more SNP are genotyped in an area of interest using, for example, Sequenom® technology, allows further analysis of LD in areas that appear to be under selection. With the information obtained from more SNP, definition of LD in the area increases, improving the ability to be able to localise causal variants using numerous statistical methods, such as iHS and XP-EHH, that have been developed to identify signatures left in the genome by selection.

As previously discussed the SNP50 BeadChip has already been used to map causal mutations for traits controlled by a single locus, and furthermore used to validate and detect selection sweeps in sheep [22, 23]. While it is perhaps surprising that only two of the regions under selection correlated with a previously identified QTL, this lends support to the widely held theory that parasite resistance is under the control of many loci with a moderate effect. New genomic approaches, including the SNP50 BeadChip, and sequencing of whole genomes [69] and transcriptomes [70], provide an opportunity to rapidly look for and find genome-wide signals of selection [71, 72].

## Conclusions

Genome wide analysis of selection signatures revealed 16 regions, which included genes involved in chitinase activity and the cytokine response. Many of the signals of selection found in this study are novel observations; further knowledge of the genes involved in gastrointestinal parasite resistance or susceptibility can only increase our understanding of the mechanisms involved.

## Methods

### Ethics statement

This study was carried out in strict accordance of the guidelines of the 1999 New Zealand Animal Welfare Act and was approved by the AgResearch’s Invermay Animal Ethics committees (Permit Numbers include: 497; 551; 593; 636; 10441; 10820).

### Selection lines

Divergent lines of Romney [6, 31] and Perendale [7] sheep were selectively bred for high and low FEC by AgResearch Ltd from 1978 and 1986, respectively (Table 3). The Perendale selection flocks were established from an initial group of 111 rams, ranked for FEC, with the high and low FEC animals mated with 148 foundation dams. The number of foundation animals for the Romney selection lines is more difficult to define, due to divergent lines from two separate locations being merged to make the final selection lines in 1993 [6]. Selection lines have now been discontinued. Animals were selected as lambs solely on the basis of FEC following a natural mixed species nematode challenge. The predominant parasites were of the *Trichostrongylus* and *Teladorsagia* genera, however *Cooperia*, *Haemonchus*, and *Nematodirus* species were also present, with other genera being less abundant [6, 7, 31]. In the 1984–89 lamb crops, of the Perendale selection lines, the natural challenge was augmented further by an artificial challenge with *H. contortus* larvae.

### Genotyping data

Animals were genotyped using the SNP50 BeadChip (Additional file 7), using high concentration DNA obtained from heparinised blood [73]. In total 180 Romney (83 high FEC animals and 97 low FEC animals) and 149 Perendale (74 high FEC animals and 75 low FEC animals) animals were genotyped. Using pedigree information, animals were chosen to be as unrelated as possible, however 66 sires and dams were also included (17 sires and 10 dams from the Romney lines, and 3 sires and 36 dams from the Perendale lines).

SNP locations for version 3.1 of the sheep genome were obtained from CSIRO (http://www.livestockgenomics.csiro.au/sheep/oar3.1.php; OARv3.1). Minor allele frequency (MAF) and call rate was calculated for each SNP. Quality control checks [74] excluded those SNP that had a call rate less than 99% and a MAF (over all animals of a breed) of less than 2%. Individual animals were removed from the analysis if there were more than 1% genotyping failure. Additionally, SNP not in Hardy-Weinberg equilibrium (HWE; p < 10^-6^) within selection line were also excluded. The Bonferroni correction was used to address the problem of multiple comparisons [75]. An experiment-wise significance level (α = 0.05) was chosen, and the number of tests was taken to be the number of SNP (n = 50,000), giving β = α /n = 1 × 10^-6^ as the test-wise significance level for HWE. This is conservative as the Bonferroni correction factor is based on independent tests. While departure from HWE may be caused by selection, it is more likely that extreme violations indicate a poorly performing SNP [76].

### Genome-wide analysis

Two single-marker tests for differentiation, F_ST_ and Peddrift, were used to distinguish signals of selection between selection lines from whole-genome data. F_ST_, which describes the proportion of variance within a species that is due to population subdivision, was calculated using Fisher’s [25] method for each breed:


Where the variance of p is computed across sub-populations, and p(1-p) is the expected frequency of heterozygotyes. The value of F_ST_ can theoretically range from 0 (no differentiation) to 1 (complete differentiation, in which subpopulations are fixed for different alleles).

Allele frequency differences at each SNP were also compared using Peddrift [11]. Peddrift calculates exact probabilities of allele frequency differences, taking into account genetic drift, founder and sampling effects. The method simulates genotypes through the actual pedigree data. Evidence of selection is shown by divergence from the expected Chi-squared (X^2^) distribution. Peddrift was run for both Romney and Perendale lines together using known pedigrees (with 2,000,000 simulations) to estimate the distribution of X^2^ under the null hypothesis of no selection. Results are expressed for each breed as a P-value for each marker.

To explore regions under selection across both breeds, the Peddrift P-values for each SNP were combined; if they have the same overall hypothesis, results from two independent tests can be combined using Fisher’s method [25], using the formula:


where p_i_ is the P-value for the i^th^ hypothesis test. The combined P-value was found by comparing *X*^2^ to a χ^2^_2k_ distribution. To reduce noise a 5 SNP moving average (WIN5) of –log_10_ of the combined P-values was used; signatures of selection are shown by SNP in a region showing low P-values. The concordance between Peddrift p-values for each SNP in Romney and Perendale was investigated by setting a p-value upper threshold of 0.01. There were 21 SNP under this threshold in both breeds, more than would be expected if there was no association in the two breeds by chance (14), suggesting that some regions had been selected in common which supports using the combined approach.

SNP were ranked using WIN5 –log_10_ (combined Peddrift P-values), and the top 0.1% of markers (n = 44) were used to determine regions under selection. The method of Kijas *et al*. [23] was used to define the boundaries of the regions; neighbouring markers were included until two consecutive markers ranked outside of the top 5%. The second marker that ranked outside of the top 5% was excluded and the position of the region determined using sheep genome assembly 3.1.

Candidate regions were annotated using Ensembl release 74 (as of 1/2014), and gene function determined using Online Mendelian Inheritance in Man (OMIM) and a literature search. Candidate genes were defined as those with a known role in the immune response. Sheep QTL were obtained from the Sheep QTLdb [38].

### Detecting signatures of selection

Fine-mapping allows further analysis of LD in areas that appear to be under selection; with the information obtained from more SNP, definition of LD in the area increases, improving the ability to be able to localise causal variants. One region on chromosome 1 (region 2) was chosen for fine-mapping with a denser set of SNP (Additional file 8), using iPLEX™ genotyping assay for the Sequenom® MassARRAY® platform. This region was chosen for fine-mapping as it contained multiple candidate genes. Selection sweep statistics were subsequently used to clarify the observed signals of selection.

All known SNP in region 2 were examined for suitability for sequencing on the Sequenom® MassARRAY® platform; these included SNP discovered on both the Solexa and 454 platforms (http://www.sheephapmap.org/genseq.php). In total 41 extra SNP were genotyped.

Linkage disequilibrium (LD) between two loci was visualised using the correlation coefficient r^2^ within each selection line using Haploview [77], with areas of strong LD indicating areas under selection.

Haplotype phase estimation was performed using fastPHASE [78]. Haplotypes were subsequently used to calculate the selection statistics EHH, XP-EHH and iHS. The EHH statistic was computed using Sweep v1.1 [79], while the iHS and XP-EHH statistics were calculated using scripts obtained from the Pritchard lab (http://hgdp.uchicago.edu/Software/). Standardized iHS (|iHS|) was calculated using the genome-wide empirical distributions, following the method of Voight *et al*. [12]. Ancestral alleles for the SNP50 BeadChip SNP were obtained from Dr Clare Gill of Texas A&M University (2009, pers. comm.), and were determined by running 11 outgroup bovid species on the SNP50 BeadChip. A cross-species megaBLAST of Sequenom® primers against *Bos taurus*, *Sus scrofa*, *Canis familiaris*, *Equus caballus* and *Homo sapiens* was used to discover ancestral alleles for the remaining SNP.

### Sequencing

Four animals were chosen for sequencing using standard amplicon sequencing (Additional file 9) with BigDye technology on an AB3730XL (Applied Biosystems). Animals consisted of one resistant and susceptible animal from each breed. Animals were selected based on homozygosity of an 11 SNP core haplotype shown by Sweep (v1.1) to be in LD (Table 2). Forward and reverse sequences were combined into contigs using Vector NTI® (Invitrogen), and consensus sequences BLASTed back against *Ovis aries CHIA* mRNA (XM_004002314.1) to search for SNP.

### Data availability

The data sets supporting the results of this article are included within the article (and its additional files).

## Electronic supplementary material

Additional file 1: **Linkage disequilibrium (LD), as shown by r** ^**2**^ **, for region 2 (OAR1:87384757–88132568) SNP in the Romney resistant selection line animals.** (PNG 162 KB)

Additional file 2: **Linkage disequilibrium (LD), as shown by r** ^**2**^ **, for region 2 (OAR1:87384757–88132568) SNP in the Romney susceptible selection line animals.** (PNG 152 KB)

Additional file 3: **Linkage disequilibrium (LD), as shown by r** ^**2**^ **, for region 2 (OAR1:87384757–88132568) SNP in the Perendale resistant selection line animals.** (PNG 159 KB)

Additional file 4: **Linkage disequilibrium (LD), as shown by r** ^**2**^ **, for region 2 (OAR1:87384757–88132568) SNP in the Perendale susceptible selection line animals.** (PNG 164 KB)

Additional file 5: **SNP in** ***CHIA*** **discovered using sequencing.** Haplotype: Sweep (v1.1) haplotype, avBV: Average FEC breeding value, Positions refer to base in *CHIA* (XM_004002314.1). (CSV 307 bytes)

Additional file 6: **All known genes in regions of interest as of 01/2014.** (CSV 4 KB)

Additional file 7: **Pedigree information and Illumina® Ovine SNP50 BeadChip data for Romney and Perendale selection line animals.** (ZIP 6 MB)

Additional file 8: **SNP used on iPLEX™ genotyping assay for the Sequenom® MassARRAY® platform.** Multiplex number; SNP ID; Amplification primer sequence; Extension primer sequence; Ancestral allele (AA). (CSV 4 KB)

Additional file 9: **PCR primers used for direct sequencing of** ***CHIA*** **exons.** (CSV 911 bytes)
