# Supplementary figures and images for: Signatures of selection in sheep bred for resistance or susceptibility to gastrointestinal nematodes
Source: BMC Genomics. 2014 Jul 30;15(1):637. doi: 10.1186/1471-2164-15-637 (PMC4124167; doi:10.1186/1471-2164-15-637)

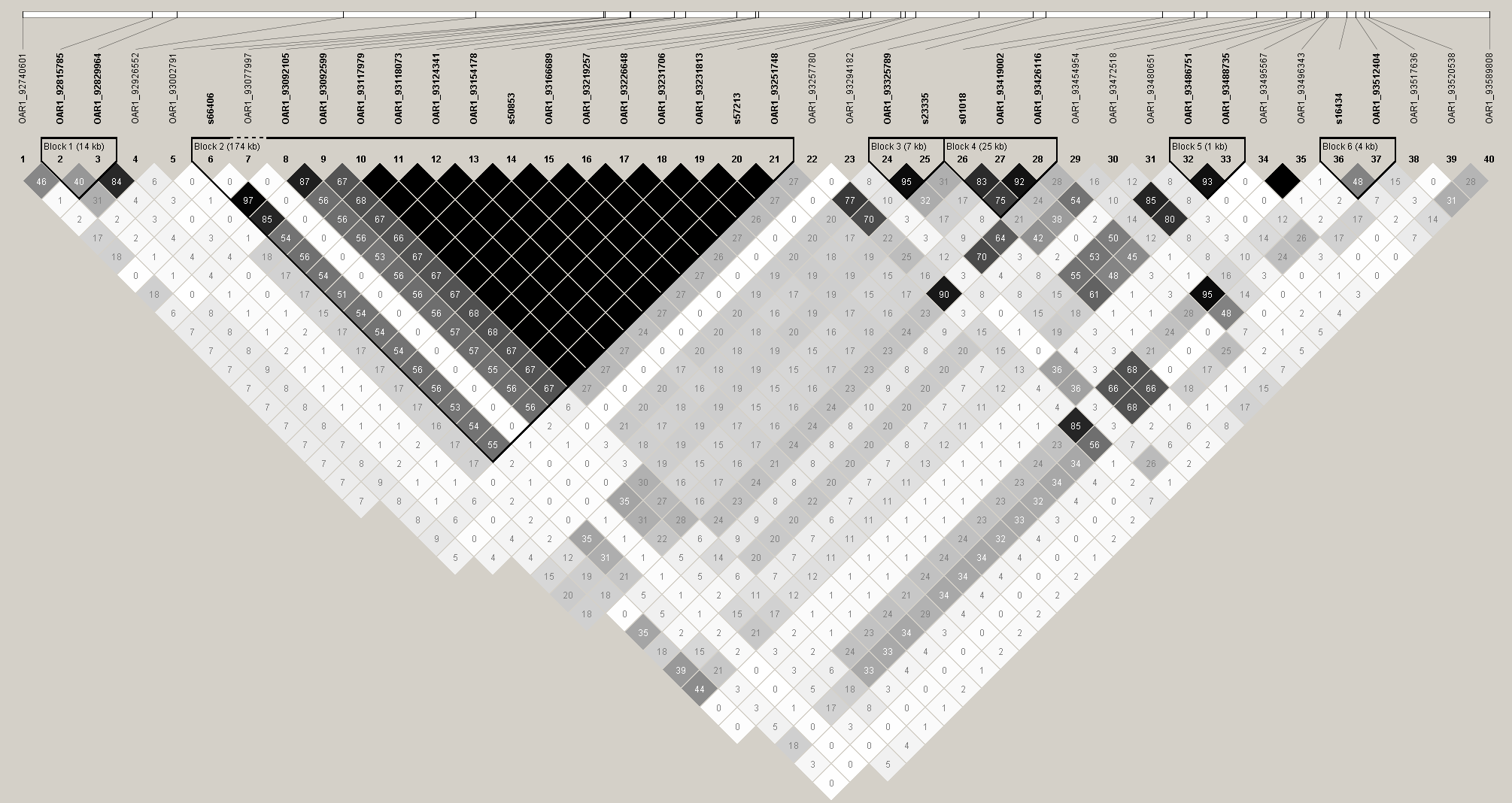

Supplement: Supplementary file 1 — Additional file 1: Linkage disequilibrium (LD), as shown by r 2 , for region 2 (OAR1:87384757–88132568) SNP in the Romney resistant selection line animals. (PNG 162 KB) [file 12864_2014_6330_MOESM1_ESM.png]

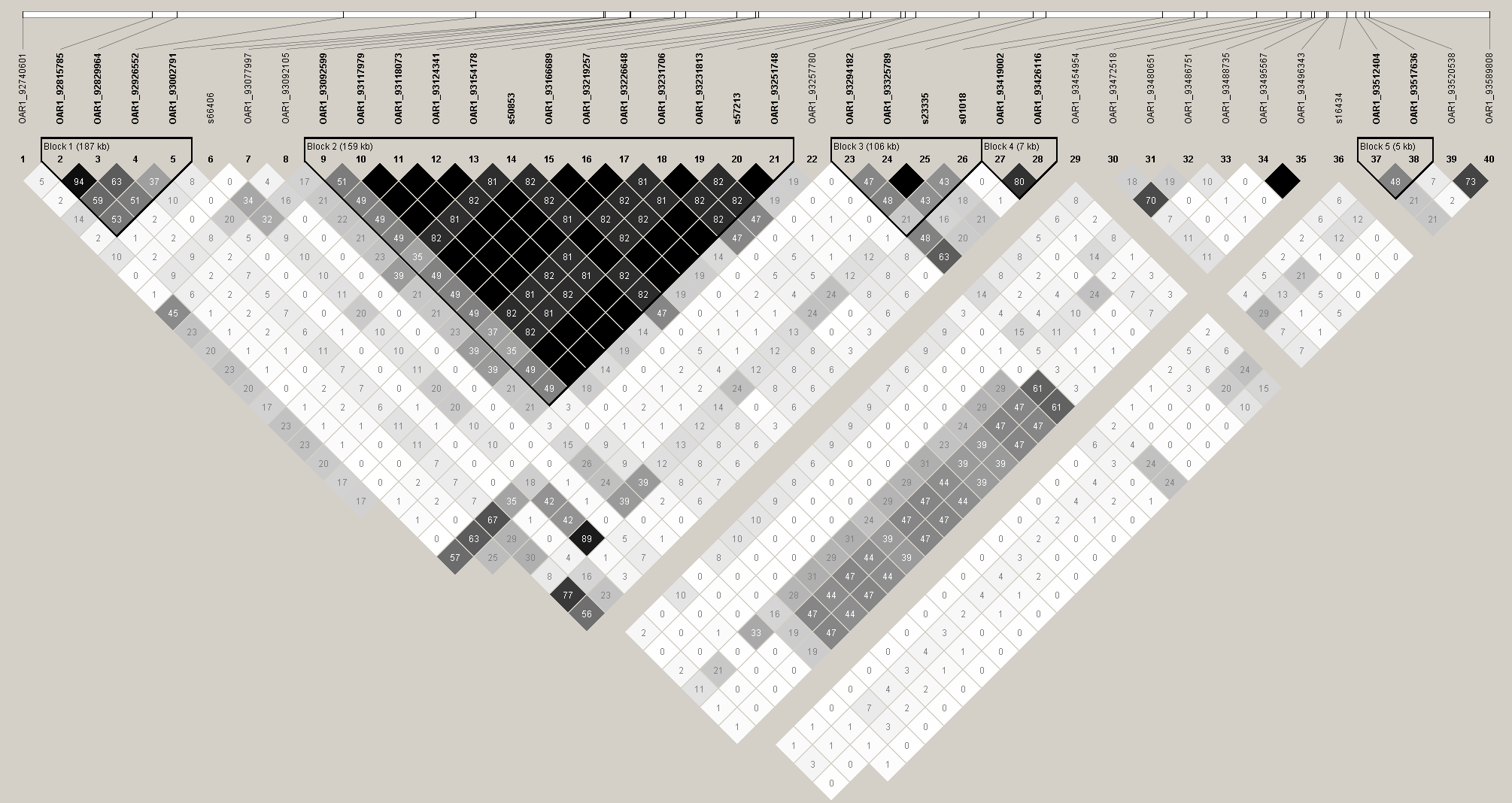

Supplement: Supplementary file 2 — Additional file 2: Linkage disequilibrium (LD), as shown by r 2 , for region 2 (OAR1:87384757–88132568) SNP in the Romney susceptible selection line animals. (PNG 152 KB) [file 12864_2014_6330_MOESM2_ESM.png]

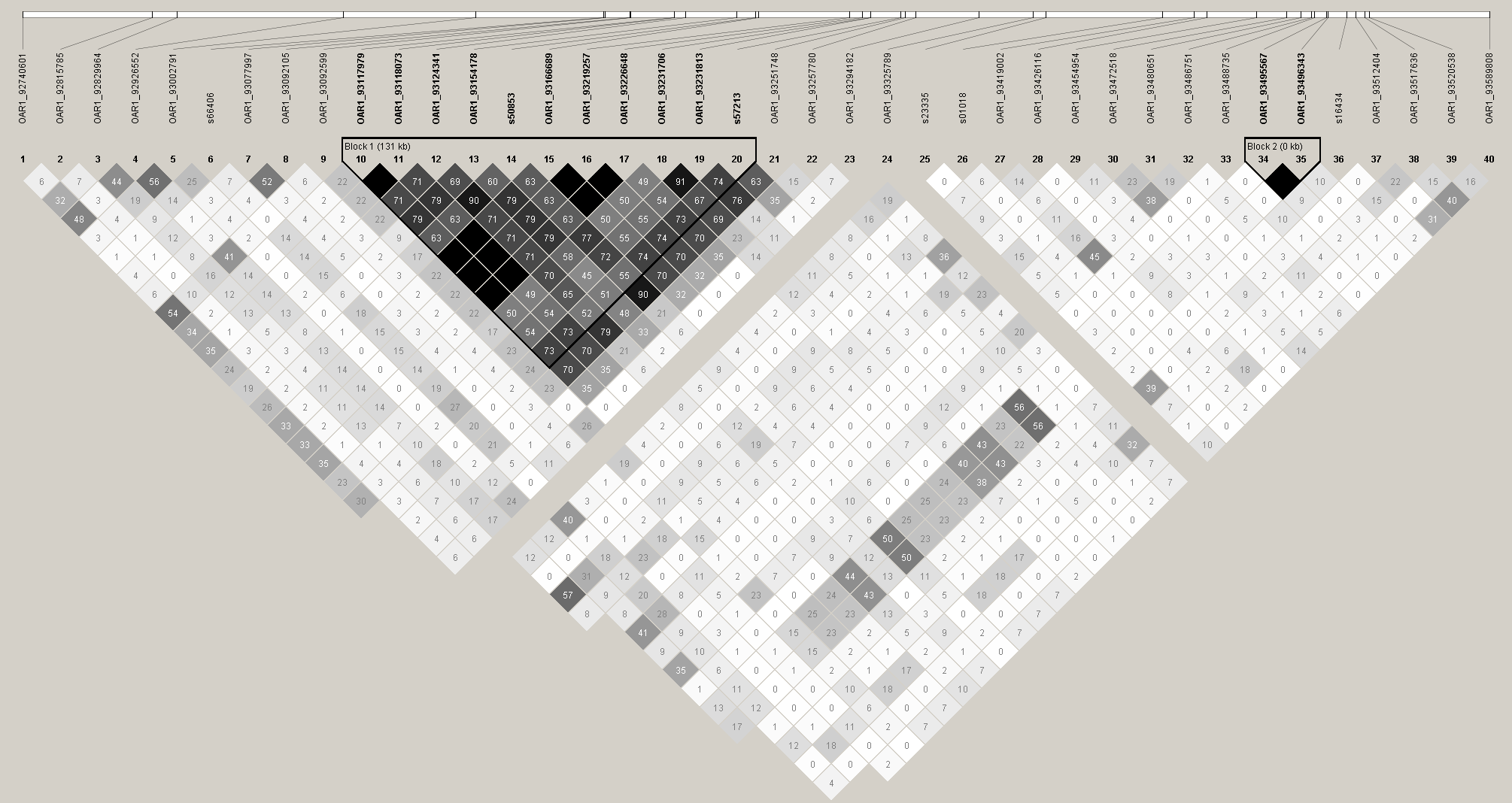

Supplement: Supplementary file 3 — Additional file 3: Linkage disequilibrium (LD), as shown by r 2 , for region 2 (OAR1:87384757–88132568) SNP in the Perendale resistant selection line animals. (PNG 159 KB) [file 12864_2014_6330_MOESM3_ESM.png]

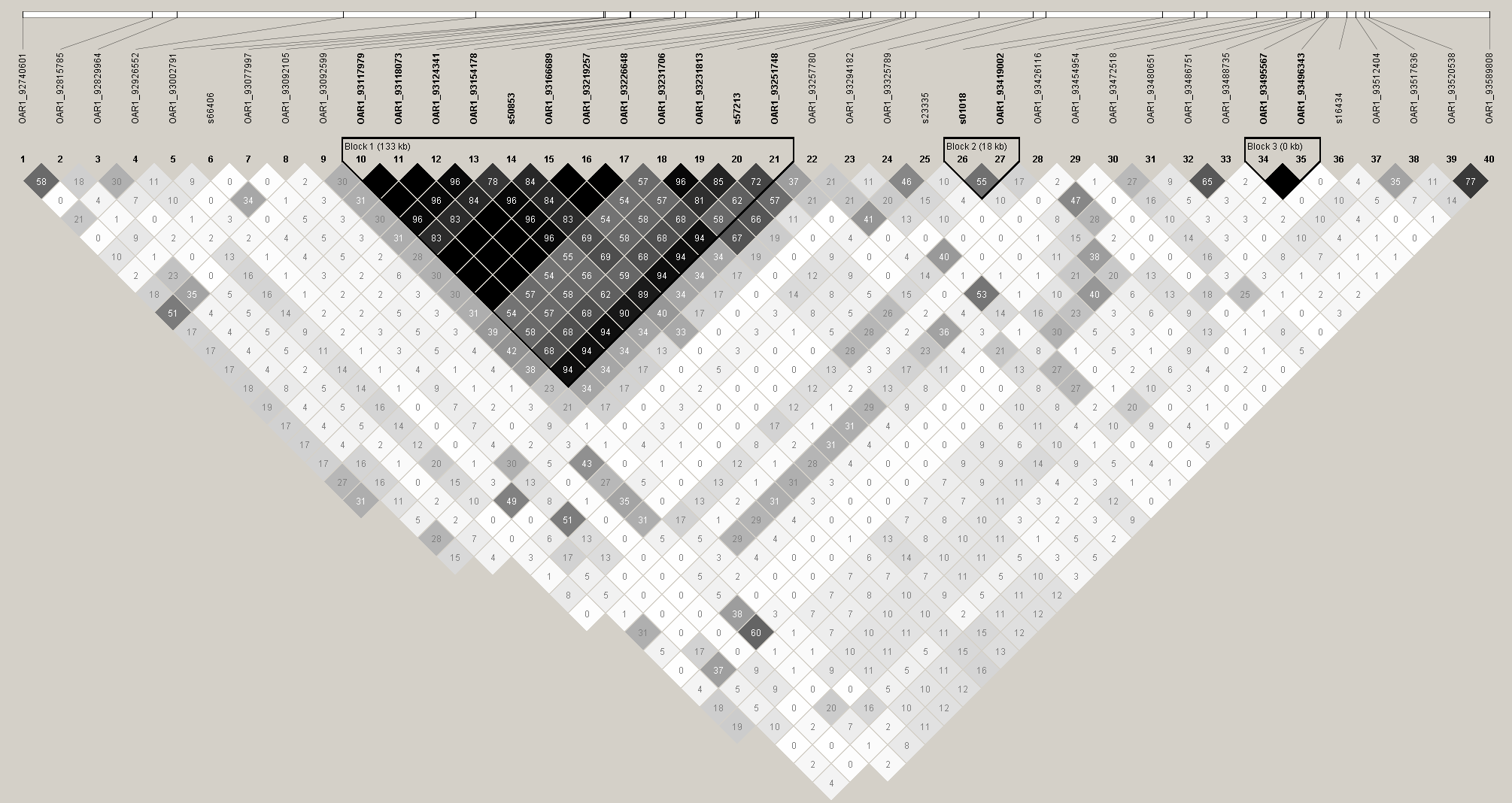

Supplement: Supplementary file 4 — Additional file 4: Linkage disequilibrium (LD), as shown by r 2 , for region 2 (OAR1:87384757–88132568) SNP in the Perendale susceptible selection line animals. (PNG 164 KB) [file 12864_2014_6330_MOESM4_ESM.png]
